# Locking Plate With or Without Cerclage Augmentation Versus Hook Plate for Neer Type II Distal Clavicle Fractures: A Single-Center Retrospective Cohort

**DOI:** 10.3390/medicina62010002

**Published:** 2025-12-19

**Authors:** Hyojune Kim, Jaeyoung Park

**Affiliations:** 1 Department of Orthopedic Surgery, Hospital of Chung-Ang University of Medicine, Dongjak-gu, Seoul 06973, Republic of Korea; 2 Department of Orthopaedic Surgery, Eulji University Hospital, Eulji University School of Medicine, Daejeon 35233, Republic of Korea

**Keywords:** distal clavicle fracture, Neer type II, hook plate, locking plate, cerclage, fragment-based cerclage augmentation

## Abstract

*Background and Objectives*: Unstable distal clavicle fractures (Neer type II) have a relatively high risk of nonunion and often require operative fixation. Hook plates are widely used, particularly when the distal fragment is small or comminuted, because they provide strong vertical stability. However, hook plates are associated with subacromial irritation, acromial wear, and the need for routine implant removal. Distal locking plates with supplementary cerclage augmentation can achieve fixation without subacromial impingement and may reduce implant-related complications. This study aimed to compare clinical and radiologic outcomes of hook plates versus locking plates with or without cerclage **augmentation** for Neer type II distal clavicle fractures. *Materials and Methods*: In this single-center retrospective cohort, adult patients with Neer type II distal clavicle fractures who underwent open reduction and internal fixation between March 2021 and August 2022, with ≥6 months of follow-up, were reviewed. Patients were allocated into two groups according to implant: hook plate (Group 1, n = 16) and distal locking plate with or without cerclage **augmentation** (Group 2, n = 26). Primary outcomes were complication rate, radiographic union, and shoulder range of motion (ROM). Secondary outcomes included pain (PVAS) and functional scores (SANE, ASES, Constant, UCLA). *Results*: Forty-two patients were analyzed (locking n = 26, hook n = 16). Groups were comparable in age (51.3 ± 16.0 vs. 54.4 ± 17.1 years), follow-up (7.0 ± 4.0 vs. 8.4 ± 4.3 months), sex distribution, smoking status, and mechanism of injury. Radiographic union was achieved in 24/26 (92.3%) patients in the locking group and 14/16 (87.5%) in the hook group; two cases of nonunion or reduction failure occurred in each group (*p* = 0.612). Final patient-reported outcomes and ROM were similar between groups (e.g., ASES 68.2 ± 15.5 vs. 64.4 ± 18.3, Constant 57.3 ± 9.5 vs. 44.9 ± 20.5; all *p* > 0.05). Forward flexion tended to be higher in the locking group (138.9 ± 28.0° vs. 113.3 ± 36.7°, *p* = 0.182), although without statistical significance. No deep infection, peri-implant fracture, or hardware failure requiring unplanned revision was observed. Subacromial wear was identified in four patients (25%) in the hook plate group, whereas no such change was observed in the locking group. *Conclusions*: Both hook plates and distal locking plates (±cerclage) provided high union rates and satisfactory functional outcomes for Neer type II distal clavicle fractures. However, hook plates were associated with subacromial wear, whereas locking plate constructs avoided subacromial complications. When distal fragment purchase is feasible—or can be supplemented with cerclage augmentation—locking plate fixation represents a reliable first-line option, with hook plates reserved for cases with minimal distal bone stock or complex comminution.

## 1. Introduction

Distal clavicle fractures represent a clinically important subset of clavicular injuries because they show a higher risk of nonunion than midshaft fractures, particularly in unstable patterns that disrupt the coracoclavicular (CC) ligaments [1,2]. Among these, Neer type II fractures frequently require operative management to restore alignment and shoulder function. Cho’s classification [3] further refines distal clavicle fracture patterns based on the relationship to the CC ligaments and fragment morphology and has been increasingly applied in surgical decision-making.

Hook plates have long been a popular choice for unstable Neer type II fractures, especially when the distal fragment is small or markedly comminuted. By engaging the undersurface of the acromion, hook plates provide strong vertical stability and help maintain reduction in the superiorly displaced medial fragment even when screw purchase in the lateral fragment is limited. However, several reports have emphasized implant-related complications, including subacromial irritation, painful stiffness, acromial erosion, and peri-implant fracture, as well as the need for routine plate removal once union is achieved [4,5,6].

The development of anatomically contoured distal clavicle locking plates has expanded the indications for plate fixation in distal clavicle fractures. When the distal fragment is sufficiently large to accept multiple screws, precontoured locking plates provide stable fixation and have become one of the preferred options for unstable distal patterns such as Cho type IIA, IIB, and IIC/ID fractures [7]. Prior biomechanical and clinical work has suggested that optimal stability requires at least four distal screws, and that bone mineral density and cortical thickness are greatest approximately 2 cm proximal to the lateral edge of the clavicle [7]. When the distal fragment is shorter than about 2.5 cm or severely comminuted, achieving this screw density becomes difficult, particularly in osteoporotic bone [8].

To address these limitations, various supplemental CC fixation techniques—including cerclage wiring and cable augmentation—have been introduced to improve vertical stability when locking plates alone cannot secure small or comminuted distal fragments. CC augmentation is especially attractive for distal clavicle fractures with CC ligament injury, because it can restore the suspensory function of the ligament complex and support reduction until union. In this setting, locking plates used in conjunction with cerclage may replicate the vertical control of hook plates while avoiding subacromial contact and its attendant complications.

Previous comparative studies have generally reported no clinically relevant differences in union rates or functional outcomes between hook plates and distal locking plates, but have noted higher complication rates with hook plates [9,10]. The similar union rates are thought to reflect that both constructs can provide adequate mechanical stability across the fracture site, whereas any observed differences in union are more likely attributable to fracture morphology (e.g., distal fragment size and comminution), bone quality, and patient-related risk factors such as smoking, rather than to the implant design itself. Building on this background, we adopted a strategy in which locking plate fixation with cerclage augmentation is preferentially used even in cases with small distal fragments or substantial comminution, reserving hook plates for only the most challenging morphologies.

A recent study by Song and Kim reported that locking plate fixation with cerclage wiring yielded higher union rates and fewer complications than hook plate fixation for unstable distal clavicle fractures treated at a single tertiary center. In contrast, the present study examines a strictly defined cohort of Neer type II (IIA and IIB) fractures treated under a different, morphology-based institutional algorithm that favors distal locking plates, selectively adds cerclage augmentation in cases with borderline distal fragment size or comminution, and reserves hook plates for only the most challenging morphologies. By including patients treated with locking plates both with and without cerclage, and by providing a fragment-based analysis of construct selection and subacromial wear, our findings complement those of Song and Kim and help refine the specific indications in which hook plates may still have a role.

Therefore, the aim of this study was to compare radiologic and clinical outcomes between hook plate fixation and locking plate fixation with or without cerclage in Neer type II distal clavicle fractures. We hypothesized that locking plate constructs would achieve comparable union and functional results with fewer implant-related complications than hook plates.

## 2. Materials and Methods

### 2.1. Study Design

This retrospective comparative study was approved by the Institutional Review Board of our institution (EMC IRB No. 2022-10-006-004), and the requirement for informed consent was waived. This retrospective comparative study included consecutive adult patients (≥18 years) with unstable Neer type II distal clavicle fractures who underwent open reduction and internal fixation at our institution between March 2021 and August 2022 and had a minimum clinical follow-up of 6 months. Exclusion criteria were: (1) prior ipsilateral shoulder surgery, (2) pathologic fractures, (3) polytrauma with competing priorities that precluded standardized rehabilitation, and (4) inadequate follow-up (<6 months) or incomplete clinical records.

Patients were categorized into two groups according to the primary fixation construct: 1) Group 1 (Hook plate): patients treated with a conventional distal clavicle hook plate (n = 16), 2) Group 2 (Locking plate ± cerclage): patients treated with a precontoured distal clavicle locking plate, with or without cerclage augmentation (n = 26). In the locking plate group, cerclage augmentation was performed in 14 of 26 patients (53.8%), whereas 12 patients (46.2%) were treated with plate fixation alone. Cerclage was used selectively in cases with small or comminuted distal fragments to assist reduction and reinforce fixation.

**Figure 1:**
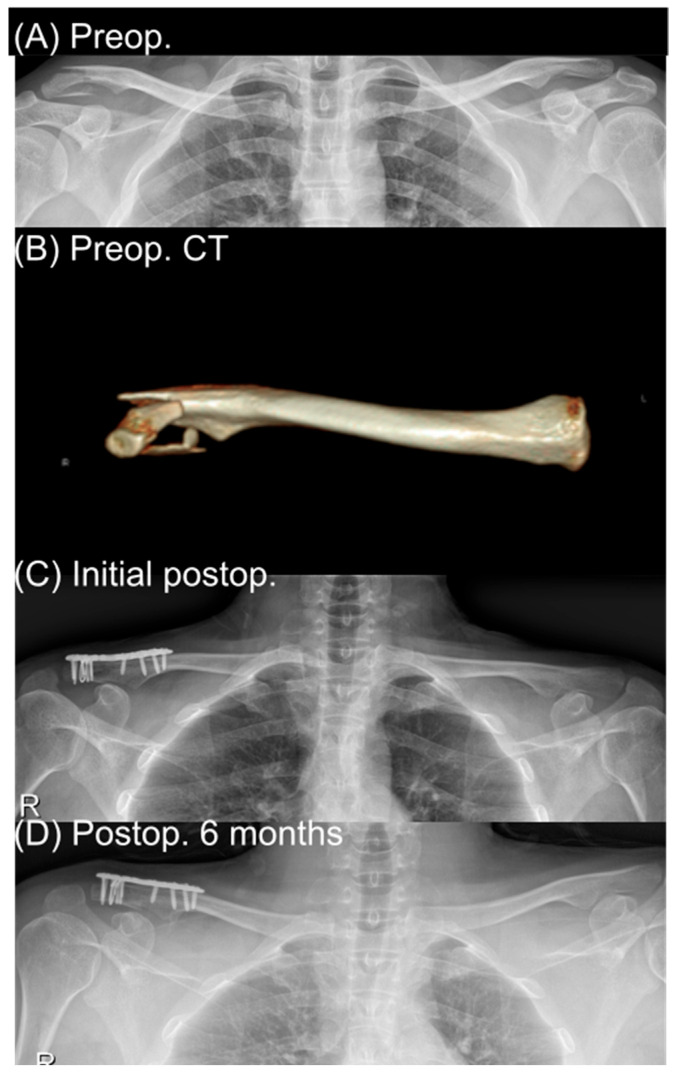
Locking plate case.

In our institutional algorithm, distal locking plates were favored when the lateral fragment was sufficiently large to accept at least four distal locking screws, typically corresponding to a fragment length ≥ 2.5 cm from the lateral edge of the clavicle. When the distal fragment was shorter than this threshold or was markedly comminuted—conditions under which anatomical plates cannot provide effective screw fixation, especially in osteoporotic bone—supplemental cerclage around the clavicle and coracoid was added to enhance vertical stability. Traditional hook plates were reserved for cases in which the distal fragment was extremely small, comminuted, or otherwise unsuitable for plate-based fixation despite augmentation (Figure 1).

### 2.2. Surgical Techniques

All procedures were performed under general anesthesia by fellowship-trained shoulder surgeons. Standard beach-chair positioning and a superior approach to the distal clavicle were used.

Locking plate ± cerclage: After careful soft tissue dissection and identification of the fracture fragments, anatomical reduction in the distal clavicle was achieved under fluoroscopic guidance. A precontoured distal clavicle locking plate was applied to the superior surface of the clavicle, and provisional fixation was obtained. When distal bone stock allowed, at least two to three distal locking screws were inserted, and efforts were made to maximize the number of distal screws.

If the distal fragment was small, osteoporotic, or comminuted, one or more 21-gauge stainless-steel cerclage loops were applied as fragment-based cerclage around the distal fragments and the proximal clavicle to assist reduction and reinforce fixation. The cerclage did not encircle the coracoid process in any case. In total, cerclage was used in 14 of 26 patients (53.8%) in the locking plate group (Figure 1).

A 31-year-old man who sustained a right distal clavicle fracture in a traffic accident was treated with distal clavicle locking plate fixation with supplementary fragment-based cerclage. (A) is a preoperative anteroposterior radiograph showing an unstable distal clavicle fracture. (B) is a three-dimensional reconstructed CT image demonstrating displacement and comminution of the distal fragment. (C) is an immediate postoperative radiograph demonstrating anatomic reduction and fixation with a precontoured distal clavicle locking plate and stainless-steel cerclage applied around the distal fragments and proximal clavicle (without encircling the coracoid). (D) is a radiograph obtained 6 months postoperatively showing maintained reduction and solid bony union.

**Figure 2:**
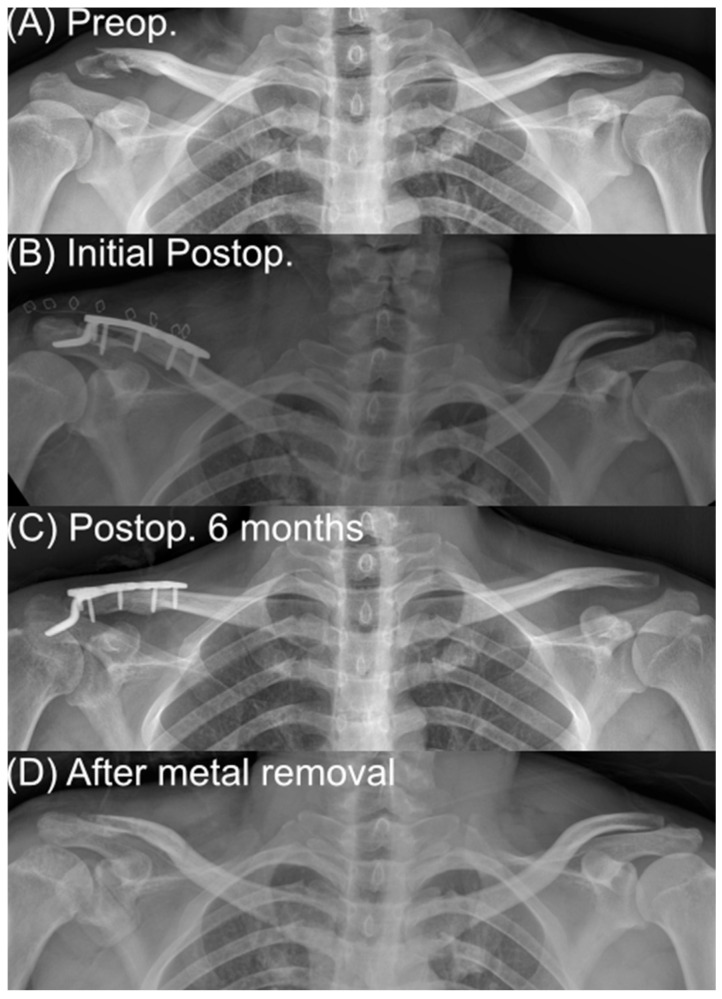
Hook plate case.

Hook plate: For hook plate fixation, a conventional distal clavicle hook plate was positioned so that the hook engaged the undersurface of the acromion. The plate was adjusted under fluoroscopy to confirm anatomic reduction, appropriate hook depth, and avoidance of gross acromial over-penetration. Screws were placed proximally and distally as permitted by bone stock. In this group, no additional coracoclavicular (CC) ligament reconstruction or augmentation (e.g., suture anchors or cerclage around the coracoid) was performed; mechanical stability was provided solely by the hook plate construct. Elective implant removal was routinely recommended after radiographic confirmation of bony union typically at 6 months postoperatively in order to reduce the risk of persistent subacromial irritation or progression of acromial erosion (Figure 2).

A 35-year-old man who sustained a distal clavicle fracture in a traffic accident was treated with hook plate fixation. (A) is a preoperative anteroposterior radiograph demonstrating an unstable distal clavicle fracture. (B) is an immediate postoperative radiograph showing restoration of alignment and fixation with a distal clavicle hook plate. (C) is a radiograph at 6 months after surgery showing bony union with the hook plate in situ. (D) is a radiograph obtained after implant removal demonstrating maintenance of fracture union and restoration of the distal clavicle contour.

### 2.3. Postoperative Rehabilitation

All patients were immobilized in an abduction sling for 2 weeks postoperatively. Gentle passive range-of-motion (ROM) exercises of the shoulder were initiated during immobilization as tolerated. Active-assisted and then active ROM exercises were progressively introduced from 6 weeks postoperatively. Strengthening and return to regular daily activities and work were permitted after radiographic confirmation of union.

### 2.4. Outcome Measures

Clinical and radiologic follow-up evaluations were scheduled at 2, 4, 6, and 8 weeks and at 3, 6, and 12 months postoperatively when possible, and at each visit the following variables were assessed. The primary outcomes were the overall complication rate (including nonunion, loss of reduction, subacromial wear, infection, peri-implant fracture, and hardware failure), radiographic union, and shoulder range of motion (ROM), comprising forward flexion (FF), external rotation (ER), and internal rotation (IR). Secondary outcomes included pain measured on the pain visual analog scale (PVAS) and functional scores, namely the Single Assessment Numeric Evaluation (SANE), American Shoulder and Elbow Surgeons (ASES) score, Constant score, and University of California Los Angeles (UCLA) shoulder score. IR behind the back was quantified by vertebral level using a 5-point ordinal scale (above T12 = 5 points, T12–L1 = 4 points, L2–L3 = 3 points, L4–L5 = 2 points, and below the sacrum = 1 point), FF and ER were measured in degrees with a goniometer, and radiographic union was defined as the presence of bridging callus or obliteration of the fracture line on standard anteroposterior clavicle radiographs.

### 2.5. Statistical Analysis

Statistical analysis was performed using SPSS version 24.0 (IBM Corp., Armonk, NY, USA). Continuous variables were compared using the independent *t*-test after confirming approximate normality; categorical variables were analyzed with Fisher’s exact test. A *p*-value < 0.05 was considered statistically significant.

## 3. Results

A total of 42 patients met the inclusion criteria: 26 in the locking plate (±cerclage) group and 16 in the hook plate group. Baseline demographic and injury characteristics were comparable between groups (Table 1). Mean age was 51.3 ± 16.0 years in the locking group and 54.4 ± 17.1 years in the hook group (*p* = 0.572). Mean follow-up duration was 7.0 ± 4.0 months versus 8.4 ± 4.3 months, respectively (*p* = 0.375). There were no significant differences in sex distribution, affected side, smoking status, or proportion of high-energy trauma between groups. All fractures were Neer type II and were subclassified as type IIA or IIB. In the locking plate group, there were 12 Neer type IIA and 14 Neer type IIB fractures, whereas in the hook plate group there were 7 Neer type IIA and 9 Neer type IIB fractures. The distribution of Neer type IIA and IIB fractures was similar between the two groups (Table 1; *p* = 1.000).

### 3.1. Clinical Outcomes

At the final follow-up, patient-reported outcomes showed no significant differences between groups (Table 2). PVAS scores were low in both cohorts (2.2 ± 1.0 in the locking group vs. 2.7 ± 2.1 in the hook group, *p* = 0.595). SANE, ASES, Constant, and UCLA scores were slightly higher in the locking plate group but did not reach statistical significance (SANE 65.6 ± 20.0 vs. 58.8 ± 24.2, *p* = 0.501; ASES 68.2 ± 15.5 vs. 64.4 ± 18.3, *p* = 0.620; Constant 57.3 ± 9.5 vs. 44.9 ± 20.5, *p* = 0.139; UCLA 28.1 ± 3.8 vs. 25.0 ± 5.0, *p* = 0.157).

ROM at the final assessment was also comparable. FF averaged 138.9 ± 28.0° in the locking plate group and 113.3 ± 36.7° in the hook plate group (*p* = 0.182). ER averaged 52.2 ± 29.1° vs. 49.0 ± 37.8° (*p* = 0.874). IR scores did not differ significantly (3.4 ± 1.5 vs. 3.0 ± 1.9, *p* = 0.684).

### 3.2. Radiological Outcomes and Complications

Radiographic nonunion or reduction failure occurred in 2 patients (7.7%) in the locking plate group and 2 patients (12.5%) in the hook plate group (*p* = 0.612), corresponding to union rates of 92.3% and 87.5%, respectively (Table 2). There were no cases of deep infection, peri-implant fracture, or catastrophic hardware failure requiring unplanned revision in either group during the follow-up period.

Subacromial acromial wear was observed in 4 patients (25%) in the hook plate group, whereas no such change was seen in the locking plate cohort (0/26 vs. 4/16, *p* = 0.016; Table 2). This finding underscores the implant-specific nature of subacromial complications associated with hook plates.

Within the locking plate cohort, 14 of 26 patients (53.8%) underwent cerclage augmentation. Subgroup comparisons between locking plate with cerclage and locking plate alone are summarized in Table 1. Briefly, radiographic union and clinical outcome measures were comparable between the two subgroups, although cerclage was more frequently applied in fractures with smaller or comminuted distal fragments.

## 4. Discussion

In our research, both hook plates and distal locking plates (with or without cerclage augmentation) yielded high union rates and satisfactory clinical outcomes under a standardized rehabilitation protocol. The primary finding is that union rates and global functional outcomes were similar between groups, whereas subacromial wear was observed only in the hook plate cohort.

Our union rates (92.3% for locking constructs vs. 87.5% for hook plates) are consistent with previously reported outcomes for operative management of unstable distal clavicle fractures, in which both plate and hook constructs generally achieve union in the majority of patients [10]. Importantly, the symmetric distribution of nonunion or reduction failure between groups suggests that, in appropriately selected cases, locking plate constructs augmented with cerclage can provide stability comparable to that of hook plates.

The present study must be interpreted in the context of the anatomical and biomechanical constraints of the distal clavicle. Previous authors have noted that the footprint of the trapezoid ligament extends approximately 2.0–2.5 cm from the lateral edge of the clavicle, and that the region of highest bone mineral density and cortical thickness lies more proximally [11,12]. In practical terms, this means that the short, ligament-bearing distal fragment frequently consists of relatively thin cancellous bone with limited capacity to hold multiple locking screws, whereas the more medial metaphyseal segment provides a more robust cortical “anchor” zone for fixation. When the distal fragment is shorter than about 2.5 cm, or heavily comminuted, mechanical purchase for multiple locking screws becomes problematic, particularly in osteoporotic bone. In addition, Shin et al. recommended inserting more than four distal screws to achieve sufficient stability, but this target is often impossible to reach in highly fragmented fractures—Erdle et al. [8] reported an average of 2.59 fragments, and Lee et al. [13] observed comminution in nearly half of distal clavicle fractures [2,14]. These morphologic realities have motivated the development of supplemental CC augmentation techniques to restore vertical stability when plating alone is insufficient [15]. Our treatment algorithm, in which locking plates are combined with cerclage in borderline cases and hook plates are reserved for the smallest or most comminuted distal fragments, was designed around these anatomic principles and likely contributed to the comparable union rates observed between constructs in our series despite their different implant-related complication profiles.

Within this framework, hook plates offer a mechanically attractive solution because they bypass distal screw purchase by leveraging the acromion to counteract vertical displacement. However, subacromial irritation, painful stiffness, and acromial erosion are well-recognized complications, often necessitating planned implant removal [16]. In our series, 25% of hook plate patients developed radiographic subacromial wear, whereas no such findings were seen in the locking plate cohort [17]. Although our sample size precludes definitive statistical conclusions about complication rates, this trend aligns with the broader literature, which has reported higher implant-related morbidity for hook plates compared with locking plates [18].

By contrast, distal locking plates with cerclage augmentation avoid subacromial contact entirely [19,20]. In cases with borderline distal fragment size, cerclage loops can effectively buttress the inferolateral fragment and restore CC stability, thereby allowing plate constructs to be used beyond the classic indication of “large distal fragment” fractures [21]. Our findings support the concept that, even in fractures with relatively small or comminuted distal fragments, a hybrid strategy of locking plate plus cerclage can yield union and functional outcomes comparable to those of hook plates, while potentially reducing the risk of subacromial complications.

From a clinical standpoint, our results suggest that construct selection can be individualized based on fragment size, comminution, and bone quality. When the distal fragment permits placement of multiple locking screws—or when cerclage augmentation can compensate for limited screw purchase—locking plate fixation appears to be a safe and effective first-line approach. Hook plates may be reserved for extreme cases where distal bone stock is insufficient for plate-based constructs despite augmentation, acknowledging the trade-off of higher implant-specific morbidity and the need for routine removal.

Our results are broadly consistent with those of Song and Kim, who also reported favorable union rates for locking plate constructs with cerclage augmentation and a higher incidence of implant-related complications with hook plates. However, the present study adds several nuances. First, by restricting the cohort to Neer type II fractures and explicitly basing implant choice on distal fragment morphology and bone quality, we were able to explore how different constructs perform within a clearly defined treatment algorithm. Second, the inclusion of locking plates both with and without cerclage augmentation allows preliminary insight into whether cerclage is necessary in all Neer type II fractures or can be reserved for cases with limited distal bone stock or comminution. Third, our detailed reporting of radiographic subacromial wear further characterizes the implant-specific risk profile of hook plates within this algorithm. Taken together, our data may be viewed as an external validation and refinement of the trends reported by Song and Kim rather than a simple replication of their study.

Several limitations need to be acknowledged in this study. First, this was a retrospective, single-center study with a modest sample size, which may limit the power to detect small differences in functional outcomes or complication rates and increases the risk of type II errors. The number of operatively treated Neer type II fractures at a single institution over the study period was limited, and larger multicenter prospective studies will be required to confirm our findings and provide more precise estimates of effect. Second, the choice of implant was at the discretion of the treating surgeons and followed an institutional algorithm rather than randomization, introducing potential selection bias. Third, although follow-up of ≥6 months is adequate to assess union and early functional recovery, longer-term outcomes—including late degenerative changes or persistent discomfort—were not fully captured. Finally, some patient-reported outcome measures and time-to-union data were incompletely documented, which may underestimate subtle differences between groups. Despite these limitations, strengths of this study include a homogeneous institutional protocol, standardized ROM measurements (including a reproducible vertebral-level scoring system for IR), and direct head-to-head comparison of hook versus locking constructs with consistent perioperative and rehabilitation pathways.

## 5. Conclusions

Both hook plate and distal locking plate fixation with or without cerclage augmentation achieved high union rates and satisfactory shoulder function in patients with Neer type II distal clavicle fractures. Two cases of nonunion or reduction failure occurred in each group, and global clinical outcomes were largely comparable. However, subacromial wear occurred only in the hook plate group, reflecting the inherent implant-related risks of subacromial hardware. Considering its ability to avoid subacromial irritation and routine implant removal while providing stable fixation—especially when combined with cerclage augmentation—distal locking plate fixation may be considered a reliable first-line option for many Neer type II distal clavicle fractures. However, given the retrospective, single-center design and modest sample size of this study, these recommendations should be interpreted with caution, and larger prospective studies are needed to confirm our findings.

## Figures and Tables

**Table 1:** Demographics.

Variable	Locking Plate (n = 26)	Hook Plate (n = 16)	*p*-Value
Age (years)	51.3 ± 16.0	54.4 ± 17.1	0.572
Follow-up (months)	7.0 ± 4.0	8.4 ± 4.3	0.375
Sex (M/F)	21/5	10/6	0.150
Side (R/L)	13/13	11/5	0.344
Smoking	10 (38.5%)	4 (25%)	0.502
High energy trauma	12 (46.2%)	7 (43.8%)	0.987
Classification (Neer type IIA/IIB), n, (%)	12 (46.2%)/14 (53.8%)	7 (43.8%)/9 (56.2%)	1.000
Cerclage augmentation, n (%)	14 (53.8%)	0 (0%)	1.000

**Table 2:** Clinical and radiological outcomes.

Variable	Locking Plate (n = 26)	Hook Plate (n = 16)	*p*-Value
PVAS	2.2 ± 1.0	2.7 ± 2.1	0.595
SANE	65.6 ± 20.0	58.8 ± 24.2	0.501
Constant	57.3 ± 9.5	44.9 ± 20.5	0.139
UCLA	28.1 ± 3.8	25.0 ± 5.0	0.157
Non-union rate	2 (7.7%)	2 (12.5%)	0.612
Forward flexion (°)	138.9 ± 28.0	113.3 ± 36.7	0.182
External rotation (°)	52.2 ± 29.1	49.0 ± 37.8	0.874
Internal rotation	3.4 ± 1.5	3.0 ± 1.9	0.684
Subacromial wear, n (%)	0 (0%)	4 (25.0%)	0.016

## Data Availability

Data is unavailable due to privacy or ethical restrictions.
